# Protein tyrosine phosphatase SHP2 promotes invadopodia formation through suppression of Rho signaling

**DOI:** 10.18632/oncotarget.4313

**Published:** 2015-06-19

**Authors:** Wan-Chen Tsai, Chien-Lin Chen, Hong-Chen Chen

**Affiliations:** ^1^ Department of Life Sciences, National Chung Hsing University, Taichung, Taiwan; ^2^ Institutue of Biomedical Sciences, National Chung Hsing University, Taichung, Taiwan; ^3^ Agricultural Biotechnology Center, National Chung Hsing University, Taichung, Taiwan; ^4^ Rong-Hsing Research Center for Translational Medicine, National Chung Hsing University, Taichung, Taiwan

**Keywords:** invadopodia, SHP2, phosphatase, invasion

## Abstract

Invadopodia are actin-enriched membrane protrusions that are important for extracellular matrix degradation and invasive cell motility. Src homolog domain-containing phosphatase 2 (SHP2), a non-receptor protein tyrosine phosphatase, has been shown to play an important role in promoting cancer metastasis, but the underlying mechanism is unclear. In this study, we found that depletion of SHP2 by short-hairpin RNA suppressed invadopodia formation in several cancer cell lines, particularly in the SAS head and neck squamous cell line. In contrast, overexpression of SHP2 promoted invadopodia formation in the CAL27 head and neck squamous cell line, which expresses low levels of endogenous SHP2. The depletion of SHP2 in SAS cells significantly decreased their invasive motility. The suppression of invadopodia formation by SHP2 depletion was restored by the Clostridium botulinum C3 exoenzyme (a Rho GTPase inhibitor) or Y27632 (a specific inhibitor for Rho-associated kinase). Together, our results suggest that SHP2 may promote invadopodia formation through inhibition of Rho signaling in cancer cells.

## INTRODUCTION

Invadopodia are F-actin-enriched membrane protrusions that localize at the ventral surface of cells and function in extracellular matrix degradation during cancer invasion and metastasis [1]. Similar structures, called podosomes, are present in several types of normal cells, such as macrophages and osteoclasts [2]. Recently, the term invadosomes has been used to include both types of structures [3]. Several key components and regulators are involved in invadopodia formation, including integrin [4, 5], actin-associated proteins, protein kinases [6, 7], proteases [8, 9], lipid kinases [10], phosphatases [11, 12], and Rho GTPases [13].

Several protein tyrosine kinases have been shown to be important for initiation of invadopodia assembly and function [6, 14, 15]. In contrast, the role of protein tyrosine phosphatases (PTPs) in the formation of invadosomes is less clear. To date, only a few PTPs have been linked to the regulation of invadosomes. PTP-1B was reported to promote invadopodia formation in breast cancer cells [16]. Additionally, PTP-ε was reported to be important for podosome stability by activating Src in osteoclasts [17]. PTP-PEST was reported to serve as a positive regulator for podosome formation in osteoclasts [18, 19], but to play a negative role in the formation of podosome rosettes in Src-transformed fibroblasts [20].

Src homolog domain-containing phosphatase 2 (SHP2), encoded by the *PTPN11* gene, is a non-receptor PTP that plays a critical role in cell proliferation [21, 22] and cell migration [23]. SHP2 acts as a positive signal transducer between receptor tyrosine kinases and the ERK pathway in mediating the cellular response to growth factors and cytokines [24]. The C-terminal Src kinase [25] and Sprouty proteins [26] have been proposed as SHP2 substrates. SHP2 promotes the activation of Src through dephosphorylation of Tyr527, which may lead to activation of the ERK signaling pathway [27]. In addition, SHP2 promotes the activation of ERK through dephosphorylating Sprouty, a negative regulator of Ras [28]. Moreover, SHP2 suppresses RhoA activity to regulate cell adhesion and migration [29–31]. Recent studies indicate that SHP2 promotes cancer cell invasion and metastasis *in vivo* [32, 33], but the mechanisms are poorly understood. It remains unclear whether SHP2 promotes tumor invasion through facilitation of invadopodia formation. Our results indicate that SHP2 promotes invadopodia formation and cell invasion through inhibition of Rho signaling in head and neck squamous cell carcinomas (HNSCC).

## RERULTS

### SHP2 plays a positive role in invadopodia formation in HNSCC cells

**Figure 1:**
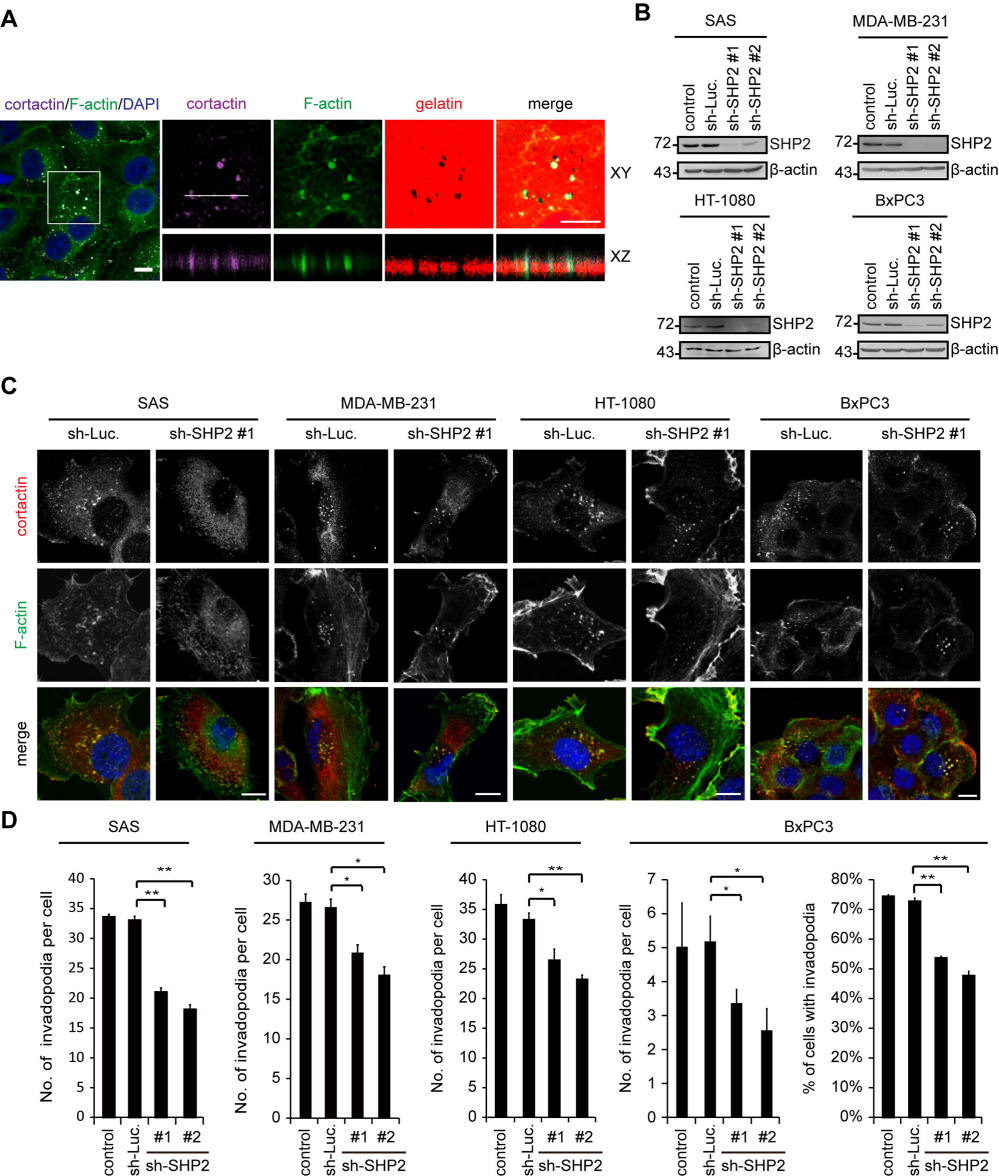
The depletion of SHP2 by shRNAs suppresses invadopodia formation in cancer cells **A.** SAS cells were seeded on Alexa Fluor 546-conjugated gelatin-coated coverslips for 54 h. The cells were fixed and then stained for F-actin, cortactin, and DAPI. The dark areas represent the areas in which the gelatin was degraded. Z stack images were obtained and reconstituted by confocal microscopy. The XY and XZ sections of the selected area containing invadopodia are shown. Scale bar, 10 μm. **B.** SAS, MDA-MB-231, HT-1080 and BxPC3 cells were infected with recombinant lentiviruses encoding shRNAs specific to luciferase (sh-Luc) or SHP2 (sh-SHP2; clones #1 and #2) and then selected in medium containing puromycin. A equal amounts of whole cell lysates was analyzed by immunoblotting with the indicated antibodies. **C.** Cells (2 × 10^5^) were grown on gelatin-coated glass coverslips for 24 h and then fixed. The fixed cells were stained for F-actin and cortactin as a marker for invadopodia. Scale bar, 10 μm. **D.** The number of invadopodia per cell and the percentage of BxPC3 cells with invadopodia out of the total number of counted cells (*n* > 100) are shown. Values (means ± s.d.) are from three independent experiments; *P < 0.05; **P < 0.001.

The role of SHP2 in invadopodia formation was examined in four different cancer cell lines, including SAS (a HNSCC cell line), MAD-MB-231 (a breast cancer cell line), HT-1080 (a fibrosarcoma cell line) and BxPC3 (a pancreatic cancer cell line). Using immunocytochemistry, F-actin dots with co-localization of cortactin (a marker for invadopodia) were considered to be invadopodia (Figure 1A). These structures were present at the ventral cell surface and were capable of degrading the underlying gelatin (Figure 1A). Invadopodia were detected in 100% of SAS cells, MAD-MB-231 cells and HT-1080 cells and 70~80% of BxPC3 cells (Figure 1D). shRNA-mediated knockdown of SHP2 decreased the number of invadopodia per cell in all four cell lines (Figure 1B–1D). The percentage of BxPC3 cells with invadopodia was also decreased by SHP2 depletion (Figure 1D).

**Figure 2:**
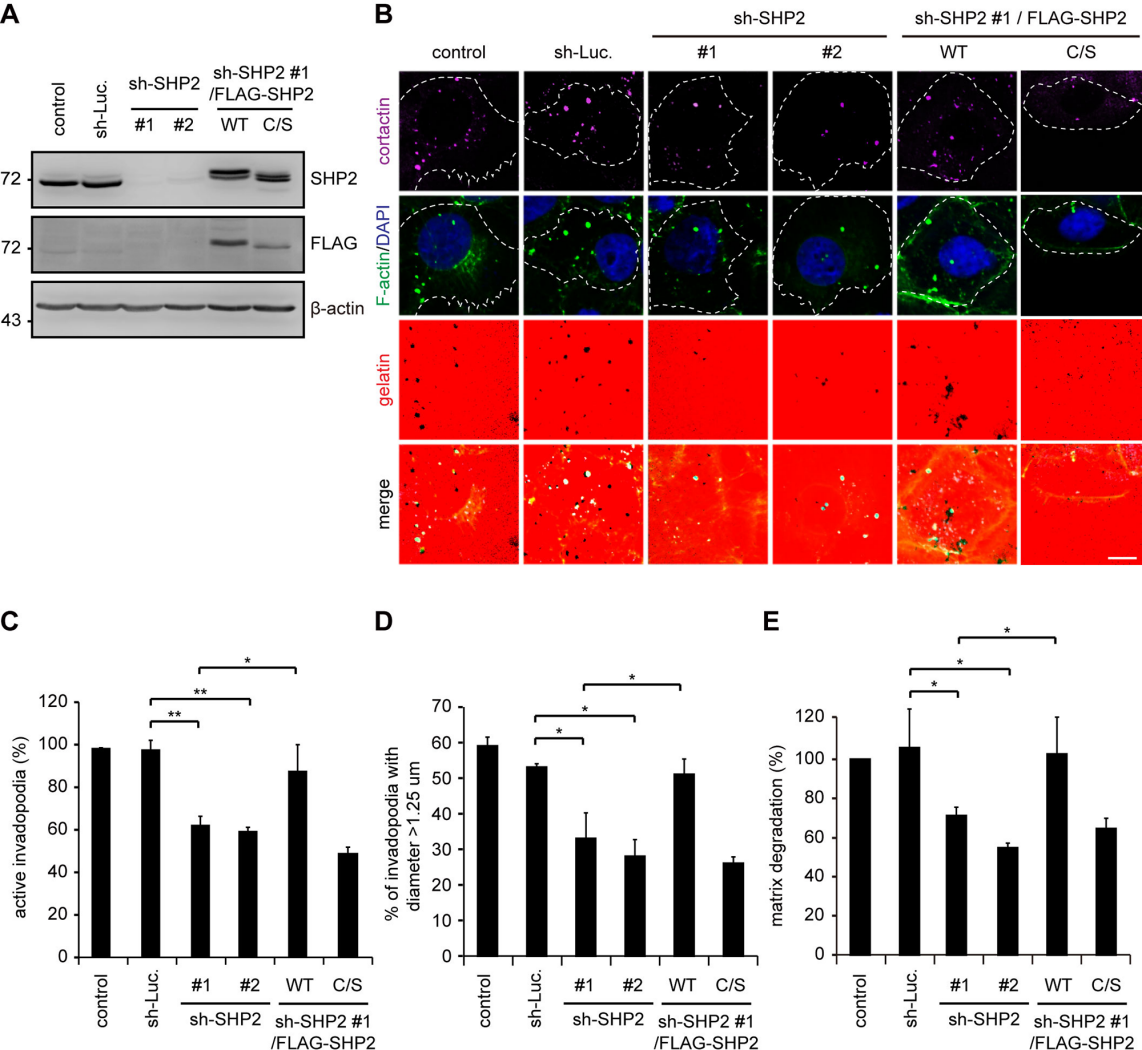
The suppression of invadopodia formation by SHP2 depletion is restored by re-expression of SHP2 but not its catalytically defective mutant in SAS cells **A.** shRNAs specific to luciferase (sh-Luc.) or SHP2 (sh-SHP2; clones #1 and #2) were stably expressed in SAS cells. Wild-type human FLAG-tagged SHP2 (FLAG-SHP2 WT) or the C459S (C/S) mutant, which is deficient in phosphatase activity, were re-expressed in the cells expressing sh-SHP2 #1. A equal amounts of whole cell lysates was analyzed by immunoblotting with the indicated antibodies. **B.** The cells were seeded on Alexa Fluor 546-conjugated gelatin-coated coverslips for 54 h. The cells were fixed and then stained for F-actin, cortactin and DAPI. Active invadopodia were defined by colocalization of F-actin and cortactin with degraded gelatin. Scale bar, 10 μm. **C.** Quantitation of active invadopodia formation in B. The number of active invadopodia per cell was determined (*n* > 150). The data are expressed as the percentage relative to the control SAS cells, which was set 100%. Values (means ± s.d.) are based on three independent experiments; *P < 0.05; **P < 0.001. **D.** Quantitation of the diameter of active invadopodia in B. The percentage of active invadopodia with a diameter over 1.25 μm out of the total number of active invadopodia (*n* > 100) is shown. Values (means ± s.d.) are based on three independent experiments; *P < 0.05; **P < 0.001. **E.** Quantitative results for the matrix degradation assay. The data are expressed as the percentage relative to the level of the control SAS cells, which was set 100%. Values (means ± s.d.) are from three independent experiments; *P < 0.05; **P < 0.001.

**Figure 3:**
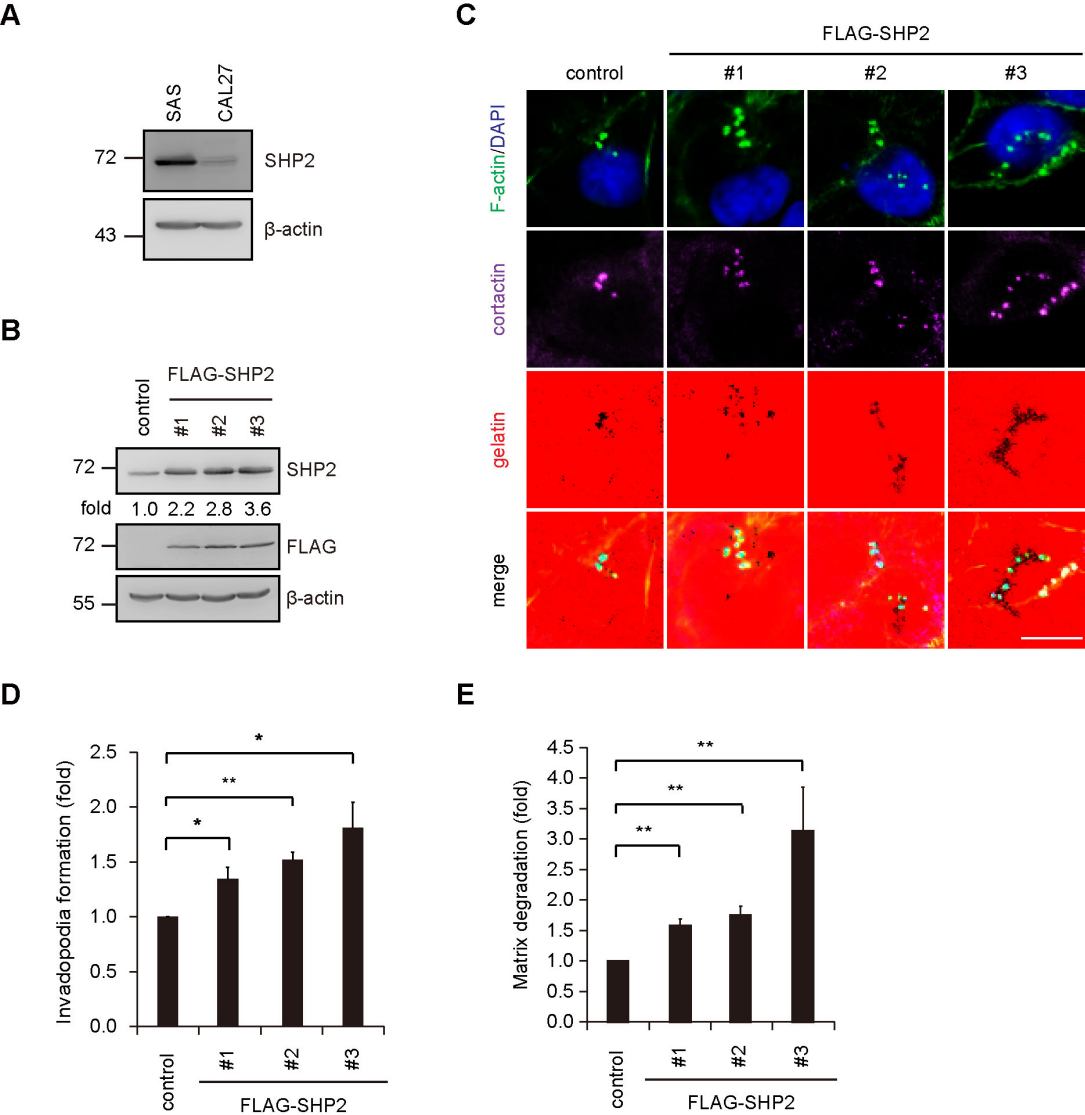
Overexpression of SHP2 in CAL27 cells, which express low levels of endogenous SHP2, increases invadopodia formation in the cells **A.** The levels of SHP2 protein were analyzed by immunoblotting with the indicated antibody in SAS and CAL27 cells. **B.** An equal amounts of whole cell lysates from CAL27 cells stably overexpressing FLAG-SHP2 (clones #1, #2 and #3) was analyzed by immunoblotting with the indicated antibody. Values (means) are from three independent experiments. **C.** Cells (4 × 10^5^) were seeded onto Alexa Fluor 546-conjugated gelatin-coated coverslips for 4 h. The cells were fixed and then stained for F-actin, cortactin and DAPI. Scale bar, 10 μm. **D.** Quantitative results for invadopodia formation. The number of invadopodia per cell was determined (*n* > 150). The data are expressed as fold relative to the control CAL27 cells. Values (means ± s.d.) are from three independent experiments; *P < 0.05; **P < 0.001. **E.** Quantitative results for the matrix degradation assay. The data are expressed as fold relative to the control CAL27 cells. Values (means ± s.d.) are from three independent experiments; *P < 0.05; **P < 0.001.

The suppression of invadopodia by SHP2-specific shRNA was, among the four cell lines examined, the most apparent in SAS cells and was restored by the re-expression of FLAG epitope-tagged SHP2 (FLAG-SHP2) but not its catalytically defective mutant (C/S mutant) (Figure 2), indicating that the phosphatase activity of SHP2 is required to promote invadopodia formation. In addition to a reduction in the number of invadopodia (Figure 2C), the size of the invadopodia was also decreased by SHP2 knockdown (Figure 2D), suggesting that SHP2 may be important for both initial assembly and maturation of invadopodia. The extent of invadopodia formation correlated with the capability of the SAS cells to degrade extracellular matrixes (Figure 2E). To further confirm the role of SHP2 in invadopodia formation, FLAG-SHP2 was stably overexpressed in HNSCC CAL27 cells, which expresses low levels of endogenous SHP2 (Figure 3). The results showed that this increased expression of SHP2 promoted the formation of invadopodia in CAL27 cells (Figure 3D) and their capability to degrade matrix proteins (Figure 3E). These data together indicate that SHP2 plays a positive role in invadopodia formation and matrix degradation of HNSCC cells.

### SHP2 is important for the invasive motility of HNSCC cells

**Figure 4:**
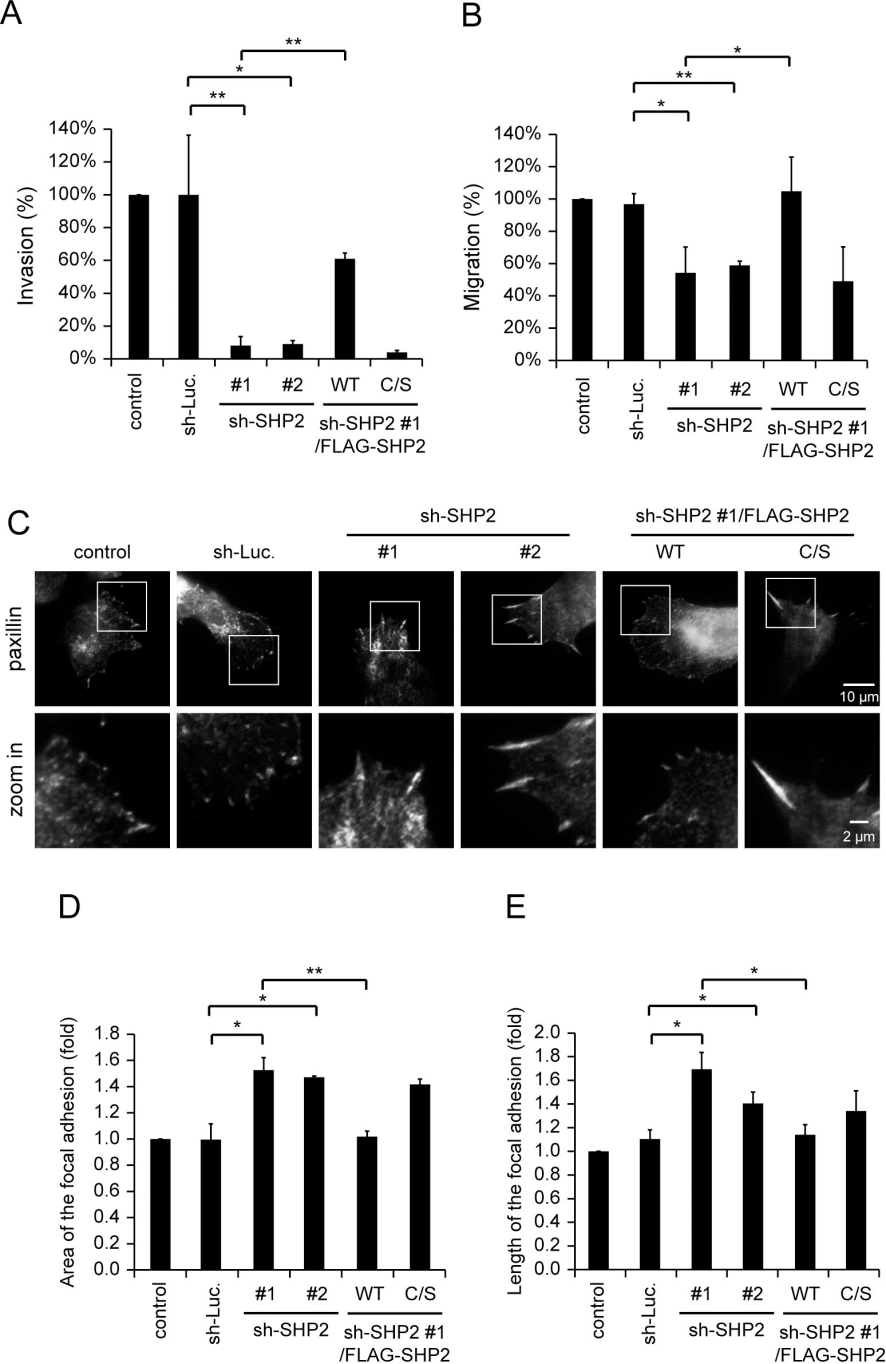
SHP2 is important for invasive cell motility **A.** Quantitative results for the Matrigel invasion assay. The data are expressed as the percentage relative to the control SAS cells, which was defined as 100%. Values (means ± s.d.) are from three independent experiments; *P < 0.05; **P < 0.001. **B.** Quantitative results for the cell migration assay. The data are expressed as the percentage relative to the level of the control SAS cells, which was defined as 100%. Values (means ± s.d.) are from three independent experiments; *P < 0.05; **P < 0.001. **C.** Cells (5 × 10^4^) were grown on gelatin-coated glass coverslips for 18 h and then fixed. The fixed cells were stained for paxillin as a marker for focal adhesions. The images were captured by total internal reflection fluorescence microscopy. **D.** Quantitative measurement of the area of the focal adhesions in C. The area of focal adhesion was measured from at least 50 cells. The data are expressed as fold relative to the control SAS cells. Values (means ± s.d.) are based on three independent experiments; *P < 0.05; **P < 0.001. **E.** Quantitative measurement of the length of the focal adhesions in C. The length of the focal adhesions was measured from at least 50 cells. The data are expressed as fold relative to the control SAS cells. Values (means ± s.d.) are based on three independent experiments; *P < 0.05; **P < 0.001.

Tumor invasion is a complicated process that requires multiple cellular functions, such as invadopodia formation, expression of matrix metalloproteases (MMPs) and cell motility. Our results show that the depletion of SHP2 suppressed ~50% of invadopodia formation and matrix degradation in SAS cells (Figure 2), but ~90% of the capability for Matrigel invasion (Figure 4A). These data suggest that other cellular activities important for cell invasion may be regulated by SHP2 as well. We also found that SHP2 is involved in the regulation of the motility of SAS cells (Figure 4B). The decrease in cell motility caused by SHP2 knockdown was correlated with increased area and length of focal adhesions in SAS cells (Figure 4C–4E). An increase in the size of focal adhesions usually represents more stable cell adhesion and less cell motility. Therefore, the profound impact of the SHP2 knockdown on the invasiveness of SAS cells may be because of its effects on both invadopodia and cell motility.

### The effect of SHP2 on the promotion of invadopodia formation is not through Sprouty2 or ERK

Sprouty2 is a negative feedback regulator of multiple receptor tyrosine kinases [26, 34]. Its dephosphorylation by SHP2 contributes to the activation of the ERK signaling pathway upon growth factor stimulation [26, 28]. In this study, we found that tyrosine phosphorylation of Sprouty2 was difficult to detect in SAS cells (Supplementary Figure S1A) and the knockdown of Sprouty2 did not affect invadopodia formation in the cells (Supplementary Figure S1B–S1D). In addition, the expression and activation of ERK was not affected by SHP2 in SAS or CAL27 cells (Supplementary Figure S2A and S2B). Invadopodia formation in both cell lines was not altered by the specific MEK inhibitor PD98059 (Supplementary Figure S2C and S2D). These data together suggest that neither Sprouty2 nor ERK was involved in invadopodia formation in HNSCC cells.

### SHP2 promotes invadopodia formation through inhibition of Rho signaling

**Figure 5:**
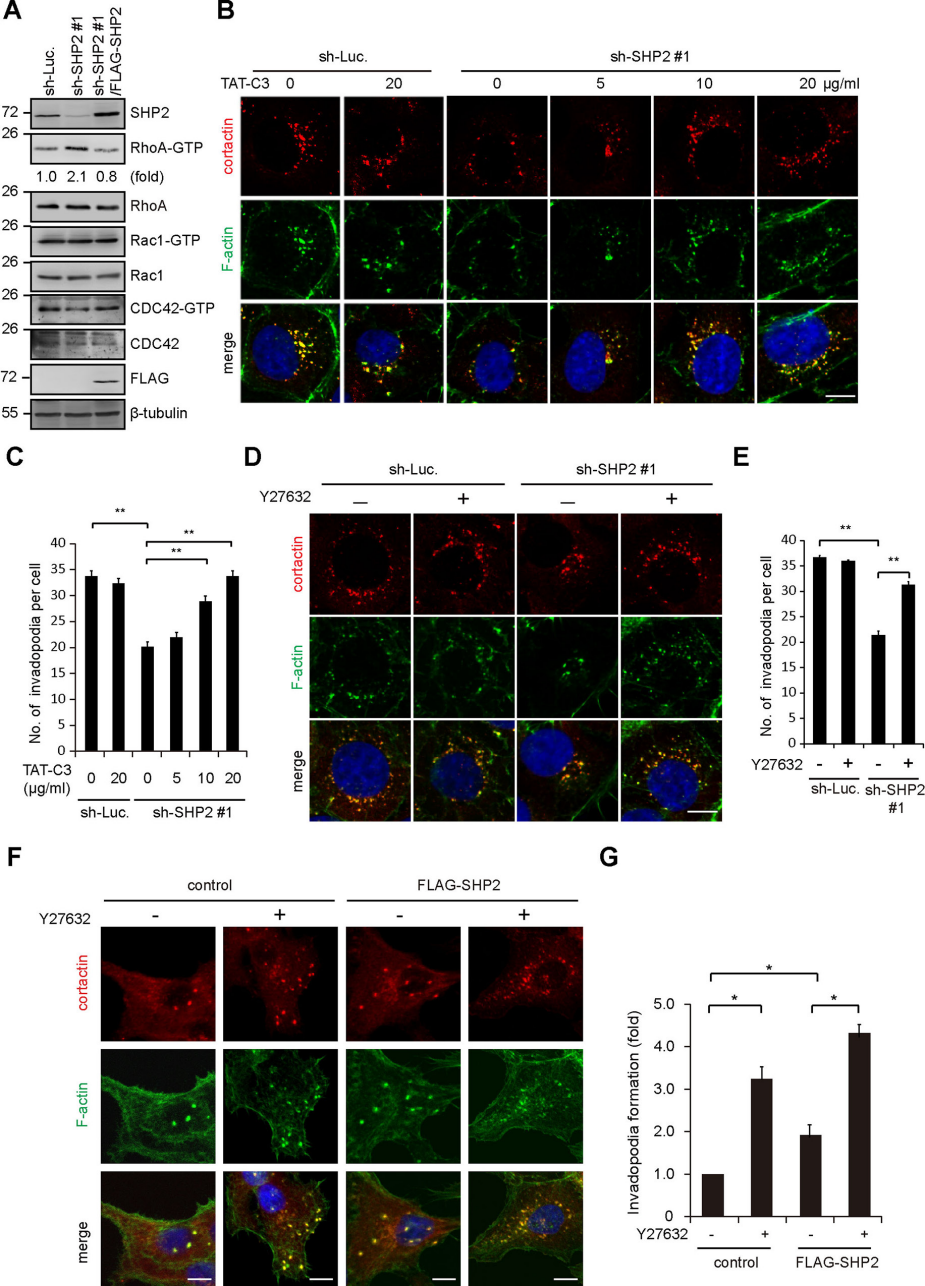
SHP2 promotes invadopodia formation by suppression of Rho activity **A.** The levels of active (GTP-bound) Rho family proteins in the cells, including RhoA, Rac1, and Cdc42, were measured. The results are representative at least three experiments. **B.** SAS cells expressing shRNA specific to SHP2 (sh-SHP2 #1) or luciferase (sh-Luc.) were grown on gelatin-coated glass coverslips for 24 h and then treated with TAT-C3 at various concentrations for 12 h and then fixed. The fixed cells were stained for F-actin and cortactin. Scale bar, 10 μm. **C.** Quantitative results of invadopodia formation in B. The number of invadopodia per cell is shown (*n* > 100). Values (means ± s.d.) are from three independent experiments; *P < 0.05; **P < 0.001. **D.** SAS cells expressing shRNA specific to SHP2 (sh-SHP2 #1) or luciferase (sh-Luc.) were grown on gelatin-coated glass coverslips for 24 h and then treated with (+) or without (−) the ROCK inhibitor Y27632 (10 μM) for 6 h and then fixed. The fixed cells were stained for F-actin and cortactin. Scale bar, 10 μm. **E.** Quantitative results of invadopodia formation in D. The number of invadopodia per cell is shown (*n* > 100). Values (means ± s.d.) are from three independent experiments; *P < 0.05; **P < 0.001. **F.** Control CAL27 cells and those stably expressing FLAG-SHP2 were grown on gelatin-coated coverslips for 24 h and then treated with (−) or without (+) the ROCK inhibitor Y27632 (10 μM) for 6 h. The cells were fixed and stained for F-actin and cortactin. Scare bar, 10 μm. **G.** Quantitative results of invadopodia formation in F. The number of invadopodia per cell was determined (*n* > 150). The data are expressed as fold relative to the control CAL27 cells. Values (means ± s.d.) are from three independent experiments; *P < 0.05; **P < 0.001.

The activation of the Rho signaling pathway has been shown to inhibit invadopodia formation [13]. In this study, we found that activation of Rho, but not Rac and Cdc42, was inversely correlated with SHP2 expression in SAS cells (Figure 5A). In addition, the suppression of invadopodia formation by SHP2 depletion was restored by application of the Clostridium botulinum C3 exoenzyme (a Rho GTPase inhibitor) (Figure 5B and 5C) or Y27632 (a specific inhibitor for Rho-associated kinase [ROCK]) (Figure 5D and 5E). Nevertheless, neither C3 exoenzyme nor Y27632 alone increased invadopodia formation in the control SAS cells (Figure 5C and 5E). These data together suggest that SHP2 depletion may lead to activation of Rho signaling, which in turn suppresses invadopodia formation. Therefore, it is possible that SHP2 promotes invadopodia formation through its suppressive effect on Rho signaling. Moreover, inhibition of ROCK by Y27632 significantly increased the formation of invadopodia in CAL27 cells (Figure 5F and 5G), indicating that suppression of ROCK is beneficial to invadopodia formation in the cancer cells with low expression levels of SHP2.

## DISCUSSION

SHP2 has been shown to be important for tumor metastasis [32, 33], but the underlying mechanism is unclear. In this study, we show that SHP2 is important for invadopodia formation in several types of cancer cell lines, particularly HNSCC cells. In addition, we demonstrate that SHP2 is also important for cell motility through its effect on focal adhesions. Therefore, it is possible that the significance of SHP2 in tumor metastasis may be, at least in part, because of its roles in promoting invadopodia formation and cell motility.

Although SHP2 has been reported to be important for ERK activation [26], this was not the case in HNSCC cells. In addition, ERK was eliminated as a possible regulator of invadopodia formation in HNSCC cells (Supplementary Figure S2). We found that the mechanism by which SHP2 promotes invadopodia formation includes suppression of the Rho signaling pathway in HNSCC cells. In fact, SHP2 has been reported to suppress Rho activity by dephosphorylation and subsequent activation of the Rho GTPase-activating proteins (GAPs) [27, 35]. However, we found that the tyrosine phosphorylation of p190RhoGAP was not modulated by SHP2 in SAS cells (Supplementary Figure S3A). In addition, the activity of Vav2, a common guanine nucleotide exchanger for Rho and Rac [36], was not affected by SHP2 in SAS cells (Supplementary Figure S3B). Therefore, the mechanism by which SHP2 suppresses the Rho activity in SAS cells remains to be investigated.

We previously reported that SHP2 suppresses the formation of podosome rosettes in Src-transformed fibroblasts [37], which raises the question of why SHP2 promotes invadopodia in HNSCC cells but suppresses podosome rosettes in Src-transformed fibroblasts. PTP-PEST was reported to serve as a positive regulator for podosome formation in osteoclasts [18, 19] but play a negative role in the formation of podosome rosettes in Src-transformed fibroblasts [20]. We speculate that the status of Src activation may determine which role SHP2 plays in such structures. In Src-transformed fibroblasts, Src is constitutively and highly active, which leads to tyrosine phosphorylation and inhibition of ROCK at Y722 [38]. Additionally, in Src-transformed fibroblasts, SHP2 counteracts the effect of Src on ROCK by dephosphorylating Y722 [39]. The activation of ROCK does not favor the formation of podosome rosettes in Src-transformed cells [40]. However, in cells where Src activity is not significantly elevated, such as SAS cells, ROCK is not phosphorylated at Y722 (Supplementary Figure S3B) and is no longer a substrate for SHP2. Under such conditions, SHP2 may target Rho GAPs rather than ROCK, which thereby suppresses Rho activity and contributes to invadopodia formation.

We showed that the suppression of invadopodia by SHP2 depletion in SAS cells was rescued by FLAG-SHP2, but not by its catalytically defective mutant (Supplementary Figure 2), indicating that the phosphatase activity of SHP2 is necessary for it to promote invadopodia formation. The tyrosine phosphorylation of several potential SHP2 substrates, such as Sprouty2, was examined (Supplementary Figure S1), but none were affected by SHP2 depletion in SAS cells. Therefore, the identities of the SHP2 substrates important for promoting invadopodia remain unknown. In addition, there is no evidence that SHP2 is localized to invadopodia, rendering it possible that the effect of SHP2 on invadopodia is indirect. Our data indicate that SHP2 regulates the formation of invadopodia through its effect on Rho signaling.

## MATERIALs AND METHODS

### Materials

Polyclonal anti-Cdc42, anti-cortactin (H-191), anti-MT1-MMP (L-15) and anti-ERK1 (K-23) antibodies and monoclonal anti-SHP2 (B-1), anti-RhoA (26C4), anti-ROCK II (H-85) and anti-β-tubulin (D-10) antibodies were purchased from Santa Cruz Biotechnology (Santa Cruz, CA). Polyclonal anti-ERK1/2 pT202/Y204 antibody was purchased from Cell Signaling Technology (Beverly, MA). Monoclonal anti-phosphotyrosine (4G10), anti-paxillin, anti-RhoGAP p190 (for immunoblotting) and anti-Rac1 antibodies and Matrigel were from BD Transduction Laboratories (San Jose, CA). Monoclonal anti-FLAG, anti-β-actin (AC-15), gelatin and protein A-Sepharose beads were from Sigma-Aldrich (St Louis, MO). Monoclonal anti-SPRY2 (M01) was purchased from Abnova. Roswell Park Memorial Institute 1640 (RPMI 1640), Dulbecco's modified Eagle's medium (DMEM), and Zeocin were from Life Technologies-Invitrogen (Carlsbad, CA). Polyclonal anti-MMP9 antibody, monoclonal anti-MMP2 and anti-RhoGAP p190 (D2D6) (for immunoprecipitation) antibodies, puromycin, PD98059 and Y27632 were from EMD Millipore (Billerica, MA). Polyclonal anti-ROCK II pY722 antibody was prepared as described previously [38].

### Plasmids

The plasmids pFLAG-CMV2-human SHP2 WT and C459S were kindly provided by D.-L.Wang (Tzu Chi University, Hualien, Taiwan). The plasmids pLKOAS2.zeo-FLAG-SHP2 WT and C459S were constructed in our laboratory. The plasmid pTAT-His-TAT-C3 was provided by Z.-F. Chang (National Yang-Ming University, Taipei, Taiwan).

### Cell culture and transfection

SAS cells and CAL27 cells were kindly provided by Muh-Hwa Yang (National Yang-Ming University, Taipei, Taiwan). HT-1080 cells were kindly provided by Meng-Hsiao Meng (National Chung Hsing University, Taichung, Taiwan). MDA-MB-231 cells were kindly provided by Ho Lin (National Chung Hsing University, Taichung, Taiwan). BxPC3 cells were kindly provided by Chia-Ron Yang (National Taiwan University, Taipei, Taiwan). SAS cells, HT-1080 cells and MDA-MB-231 cells were maintained in DMEM supplemented with 10% FBS and CAL27 cells were maintained in DMEM supplemented with 10% serum. BxPC3 cells were maintained in RPMI-1640 supplemented with 10% serum.

### Lentivirus production and infection

The lentiviral expression system was provided by the National RNAi Core Facility, Academia Sinica, Taiwan. For FLAG-SHP2 expression, human FLAG-SHP2 cDNA was amplified by polymerase chain reaction and subcloned in frame to the NheI and EcoRI site of the pLKO-AS2-zeo vector. The pLKO-AS1-puro plasmids, which express shRNAs, were obtained from the National RNAi Core Facility, Academia Sinica. The target sequences for human SHP2 are 5′-GCAGTTAAATTGTGCGCTG TA-3′ (#1) and 5′-GCTGAAATAGAAAGCAG AGTT-3′ (#2). The target sequences for human SPRY2 are 5′-CCCTCTGTCCAGATCCATAAG-3′ (#1), 5′-GGGTGTTATGACCGGGTTAAC-3′ (#2) and 5′-CTTTGCTGTTTGCGGTGAAAT-3′ (#3). To produce lentiviruses, HEK293T cells were co-transfected with pCMV-ΔR8.91 (2.25 μg), pMD.G (0.25 μg), and pLKO-AS1-puro-shRNA (or pLKO-AS2-zeo-FLAG-SHP2; 2.5 μg) using Lipofectamine. After 3 days, media containing lentivirus particles were collected and stored at −80°C. Cells were infected with lentiviruses encoding shRNAs or FLAG-SHP2 for 24 hours and subsequently selected in growth medium containing 0.5–1 mg/ml puromycin or 50 μg/ml zeocin.

### Immunoprecipitation and immunoblotting

Cells were lysed in 1% Nonidet P-40 lysis buffer (1% Nonidet P-40, 20 mM Tris-HCl, pH 8.0, 137 mM NaCl, 10% glycerol, and 1 mM Na_3_VO_4_) containing protease inhibitors (1 mM phenylmethylsulfonyl fluoride, 0.2 trypsin inhibitory units/ml aprotinin, and 20 μg/ml leupeptin). The lysates were centrifuged for 10 min at 4°C to remove debris, and the protein concentrations were determined using a Bio-Rad protein assay (Hercules, CA). For immunoprecipitation, aliquots of lysates were incubated with l μg antibody for 2.5 h at 4°C. Immunocomplexes were collected on protein A Sepharose beads. For monoclonal antibodies, protein A-Sepharose beads were coupled with rabbit anti-mouse immunoglobulin G (1 μg) before use. The beads were washed three times with 1% NP-40 lysis buffer, boiled for 3 min in sodium dodecyl sulfate (SDS) sample buffer, subjected to SDS-polyacrylamide gel electrophoresis, and transferred to nitrocellulose (Schleicher and Schuell, Inc., Keene, NH). Immunoblotting was performed with appropriate antibodies using an Amersham Pharmacia Biotech enhanced chemiluminescence system for detection. Chemiluminescent signals were detected and quantified using a luminescence image system (LAS-3000 and LAS-4000 mini, Fujifilm).

### Matrix degradation assay

Alexa Fluor 546-conjugated gelatin was prepared according to the manufacturer's instructions (Invitrogen). Cells were plated on glass coverslips coated with 2 mg/ml Alexa Fluor 488-conjugated fibronectin or gelatin. After various durations of time, the cells were fixed and stained for F-actin and nuclei. The areas in which Alexa Fluor 488-conjugated matrix proteins were degraded were measured using Photoshop (CS6 Extended; Adobe Systems, Inc.). A total of 10 random fields equivalent to 2 mm^2^ were measured.

### Matrigel invasion assay

24-well transwell chambers (Costar) separated by a membrane with 8-μm pores were coated with 100 μl Matrigel (~2.7 mg/ml). The lower chamber was loaded with 750 μl DME with 10% serum. The cells (5 × 10^4^) were added to the upper chamber in 250 μl of serum-free medium. After 24 h, the cells that had migrated through the Matrigel were fixed by methanol, stained by Giemsa stain, and counted.

### Immunofluorescent staining, laser-scanning confocal fluorescent microscopy and total internal reflection fluorescence microscopy

For immunofluorescent staining, cells were fixed by 4% paraformaldehyde (PFA) in phosphate buffered saline (PBS) for 30 min at room temperature and permeabilized with 0.1% Triton X-100 in PBS for 10 min at room temperature. To stain paxillin in cells, cells were fixed by cold 97% methanol and 3% PFA for 10 min at −20°C and permeabilized with 0.1% Triton X-100 in PBS for 10 min at room temperature. The fixed cells were stained with primary antibodies at room temperature 3 hours followed by rhodamine-or Cy5-conjugated secondary antibodies (Invitrogen) incubated overnight at 4°C. The primary antibodies used for immunofluorescent staining in this study were monoclonal anti-paxillin (1:200) and polyclonal anti-cortactin (1:100). Alexa Fluor 488-conjugated phalloidin (Invitrogen) was used to stain actin filaments. Coverslips were mounted in Anti-Fade DAPI-Fluoromount-G (SouthernBiotech) and viewed using a laser-scanning confocal microscope image system (LSM 510; Carl Zeiss) with a 63 × Plan-Apochromat (NA 1.2 W Korr; Carl Zeiss).

The areas in which Alexa Fluor 488-conjugated matrix proteins were degraded were measured using Photoshop (CS6 Extended; Adobe Systems, Inc.). A total of 10 random fields equivalent to 2 mm^2^ were measured. The areas and length of focal adhesions were measured using Photoshop (CS6 Extended; Adobe Systems, Inc.) and Image J 1.47n (National Institutes of Health). For total internal reflection fluorescence microscopy, the coverslips were viewed using an inverted Zeiss microscope (Axio Observer D1) with α Plan-Fluor 100X/1.45 III objective.

### Small GTPase activity assay

GTP-bound RhoA in whole-cell lysates was pulled down by immobilized GST-Rhotekin-Ras-binding domain. GTP-bound Rac and Cdc42 in whole-cell lysates were pulled down by immobilized GST-p21-activated kinase-Ras-binding domain. The washed complexes were analyzed by immunoblotting with an antibody specific to RhoA, Rac1, or Cdc42.

### Purification of His-tagged TAT-RhoV14 and TAT-C3

Purification of His-tagged TAT-C3 was performed as described previously [39]. His-tagged TAT fusion proteins were expressed in BL21 (DE3) *Escherichia coli* by isopropyl β-D-thiogalactopyranoside induction. The bacteria were lysed in lysis buffer (6 M Urea, 20 mM Tris, pH 7.9, 500 mM NaCl, and 5 mM imidazole), and His-tagged TAT fusion proteins were immobilized on Ni-nitrilotriacetic acid beads. The complexes were washed once with lysis buffer and twice with washing buffer (20 mM Tris, pH 7.9, 500 mM NaCl, and 20 mM imidazole) and were then eluted with elution buffer (20 mM Tris, pH 7.9, 500 mM NaCl, and 1 M imidazole). The eluted proteins were dialyzed three times with 200 ml of 5% glycerol in PBS at 4°C for 15 min and stored at −80°C.

### Cell migration assays

Mouse Type IV collagen was purchased from Gibco BRL (Gaithersburg, MD). Migration experiments were carried out in a Neuro Probe 48-well chemotaxis chamber (Cabin John, MD). The lower chamber was loaded with 30 μl serum-free medium with type IV collagen. The cells (10^4^) were added to the upper chamber in 50 μl of serum-free medium per well. The lower and upper chambers were separated by a polycarbonate membrane (8 μm pore size, Poretics, Livermore, CA). Cells were allowed to migrate on 10 μg/ml collagen for 6 hours at 37°C in a humidified atmosphere containing 5% CO_2_. The membrane was fixed in methanol for 8 minutes and stained with modified Giemsa stain (Sigma, St Louis, MO) for 1 hour. Cells on the upper side of the membrane were then removed mechanically. Cells on the lower side of the membrane were enumerated using a light microscope at × 200 magnification. One data point comprised the average number of cells in five random wells.

### Statistical analysis

The data were analyzed by Student's *t*-test. Differences were considered to be statistically significant at *P* < 0.05 or *P* < 0.001.

## SUPPLEMENTARY FIGURES



## Abbreviation

SHP2: Src homolog domain-containing phosphatase 2
PTP: protein tyrosine phosphatase
HNSCC: head and neck squamous cell carcinoma
shRNA: short-hairpin RNA
MMP: matrix metalloprotease
GAP: GTPase-activating protein
ROCK: Rho-associated kinase
