# Supplementary material for: Protein tyrosine phosphatase SHP2 promotes invadopodia formation through suppression of Rho signaling
Source: Oncotarget. 2015 Jun 19;6(27):23845–56. doi: 10.18632/oncotarget.4313 (PMC4695156; doi:10.18632/oncotarget.4313)
Supplement: Supplementary file 1 [file oncotarget-06-23845-s001.pdf]

SUPPLEMENTARY FIGURES

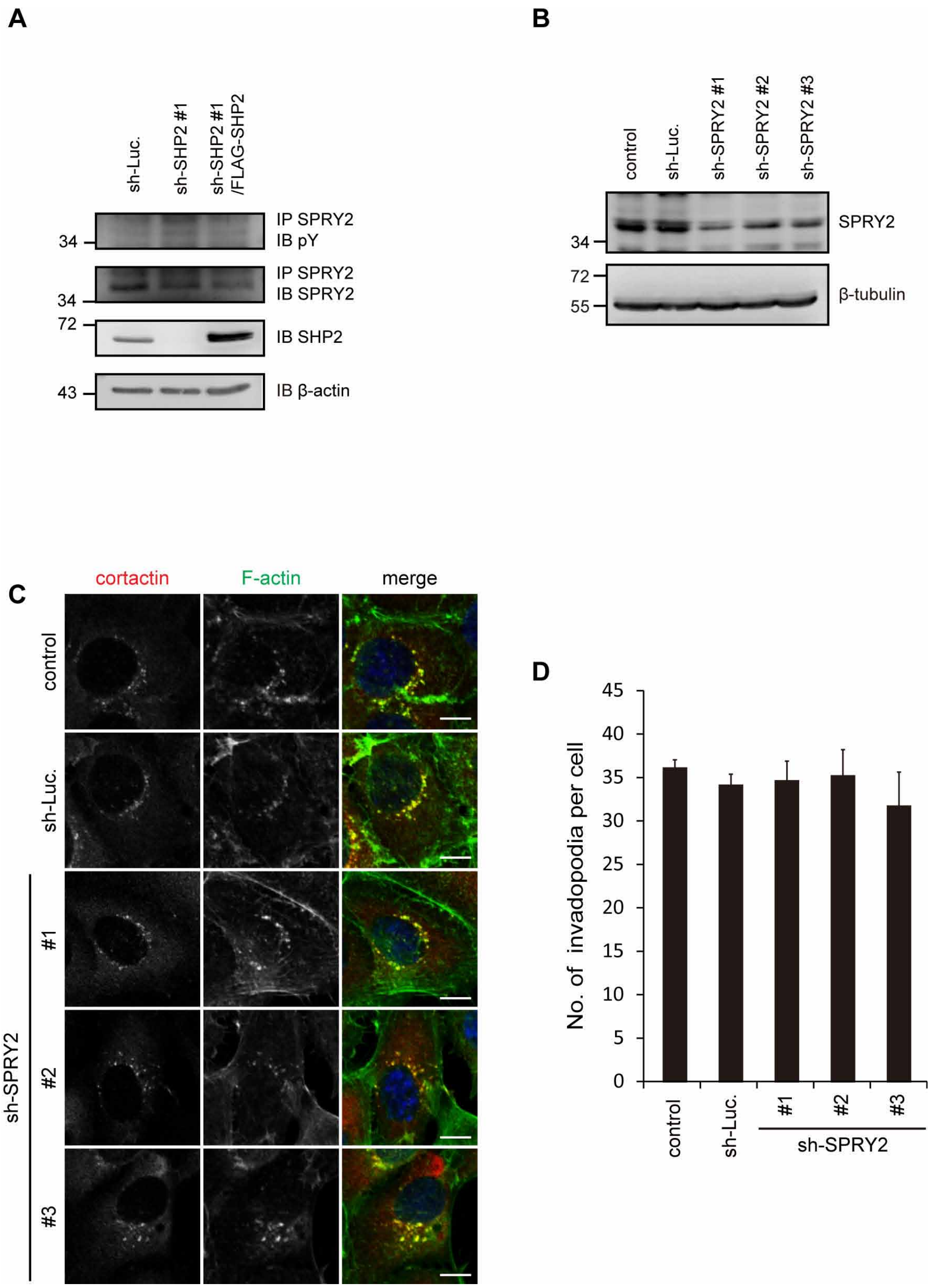

Supplementary Figure S1: Sprouty2 is not involved in invadopodia formation in SAS cells. A. Endogenous sprouty2 (SPRY2) proteins from SAS cells were immunoprecipitated (IP) by anti-SPRY2. The immunocomplexes were subjected to immunoblotting with anti-phosphotyrosine or anti-SPRY2. **B.** shRNAs specific to SPRY2 (sh-SPRY2 clones #1, #2 and #3) or luciferase (sh-Luc.) were stably expressed in SAS cells. Equal amounts of cell lysates from SAS cells were analyzed by immunoblotting with the indicated antibodies. **C.** Cells (2x10^5) were grown on gelatin-coated glass coverslips for 24 h and then fixed. The fixed cells were stained for F-actin and cortactin as a marker for invadopodia. Scale bar, 10 μm. **D.** Quantitative results for invadopodia formation. The number of invadopodia per cell was measured (*n* >100). The data are expressed as fold relative to the control SAS cells. Values (means ± s.d.) are from three independent experiments.

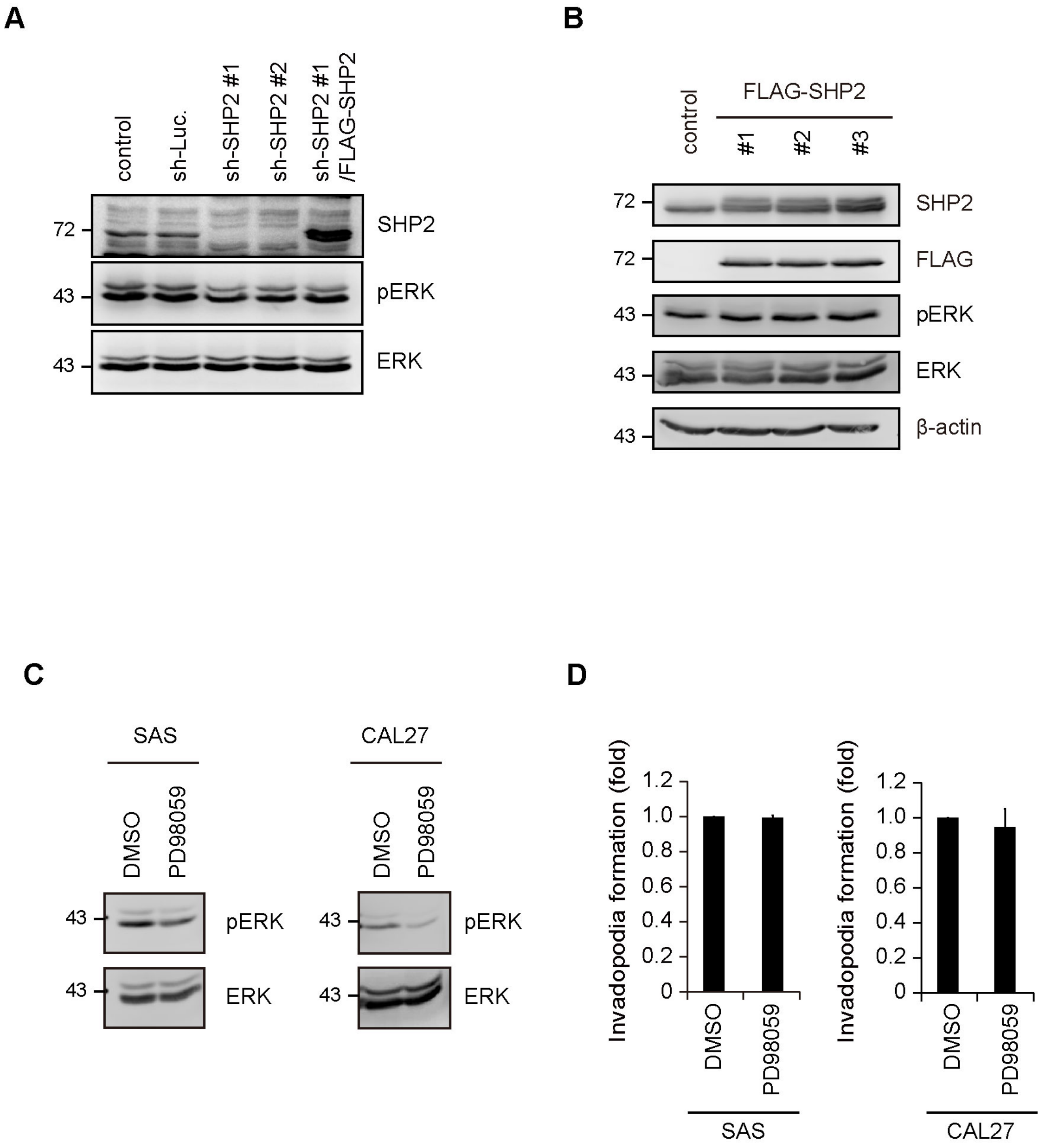

Supplementary Figure S2: ERK is not involved in invadopodia formation in SAS cells or CAL27 cells. A. shRNAs specific to SHP2 (sh-SHP2 clones #1 and #2) or luciferase (sh-Luc.) were stably expressed in SAS cells. FLAG-SHP2 was re-expressed in the cells expressing sh-SHP2 #1 (sh-SHP2 #1/FLAG-SHP2). An equal amount of cell lysates from SAS cells was analyzed by immunoblotting with the indicated antibodies. **B.** An equal amounts of whole cell lysates from CAL27 cells stably overexpressing FLAG-SHP2 (clones #1, #2 and #3) was analyzed by immunoblotting with the indicated antibody. **C.** SAS or CAL27 cells were grown for 24 h and then treated with 25 µM PD98059 (a MEK1 inhibitor) for 4 h. For the control, an equal volume of the solvent DMSO was added to the medium. An equal amount of cell lysates was analyzed by immunoblotting with the indicated antibodies. **D.** SAS or CAL27 cells were grown for 24 h and the treated with 25 µM PD98059 for 4 h. Quantitative results of the invadopodia formation are shown. The data are expressed as fold relative to the control SAS or CAL27 cells. Values (means ± s.d.) are from three independent experiments.

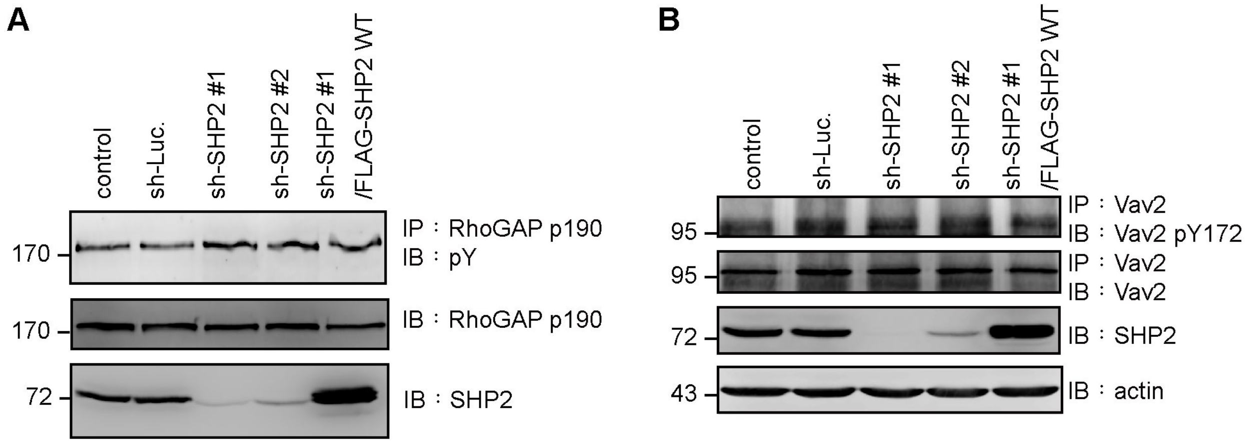

Supplementary Figure S3: SHP2-mediated suppression of the Rho activity is not through p190RhoGAP or Vav2 in SAS cells. A. shRNAs specific to SHP2 (sh-SHP2 clones #1 and #2) or luciferase (sh-Luc.) were stably expressed in SAS cells. FLAG-SHP2 was re-expressed in the cells expressing sh-SHP2 #1 (sh-SHP2 #1/FLAG-SHP2). Endogenous RhoGAP p190 proteins were immunoprecipitated (IP) by anti- p190RhoGAP. The immunocomplexes were subjected to immunoblotting with anti-phosphotyrosine. An equal amount of cell lysates was analyzed by immunoblotting with the indicated antibodies. **B.** Endogenous Vav2 proteins from SAS cells as described in (A) were immunoprecipitated (IP) by anti-Vav2. The immunocomplexes were subjected to immunoblotting with anti-Vav2 or a phospho-specific antibody to pY172. The level of Vav2 pY172 is used to indicate the guanine nucleotide exchange activity of Vav2. An equal amount of cell lysates was analyzed by immunoblotting with anti-SHP2 or anti-actin.
